# The Boston All‐in‐One Medial Patellofemoral Complex Reconstruction: A Combined Medial Patellofemoral Ligament and Medial Quadriceps Tendon Femoral Ligament Dynamic Tensioning Technique Using Allograft

**DOI:** 10.1002/atn2.70386

**Published:** 2026-10-29

**Authors:** Khalid Al‐Hourani, Xinning Li

**Affiliations:** ^1^ Frimley Knee Unit Frimley Health NHS Foundation Trust Surrey UK; ^2^ Falcon Sports Clinic London UK; ^3^ Department of Orthopaedic Sports Medicine Boston Medical Center Boston Massachusetts U.S.A.

## Abstract

Recent anatomical understanding of the medial restraints of the patella has evolved over the last decade with a current focus on the medial quadriceps tendon femoral ligament, a distinct anatomical structure to the medial patellofemoral ligament. These combine to form the medial patellofemoral complex. This technical note describes a technique to reconstruct the whole medial patellofemoral complex, replicating anatomical knowledge and allowing for dynamic graft tensioning using an allograft.

## Graphical abstract

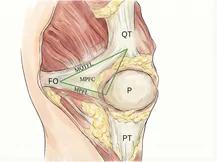

**VIDEO 1:** A narrated video depicting the medial patellofemoral ligament complex reconstruction technique on a cadaveric right knee as per the article. Video content can be viewed at https://doi.org/10.1002/atn2.70386.

Our understanding of the medial restraints to lateral patellar translation has evolved. Traditionally, the focus has been on the medial patellofemoral ligament (MPFL).^1^ However, more recent anatomical studies and subsequent data have shown an additional anatomically distinct component of the medial retinaculum arising from the femoral insertion of the MPFL and attaching to the anterior medial third of the quadriceps tendon, known as the medial quadriceps tendon femoral ligament (MQTFL).^2^, ^3^ As a result, this forms a triangular complex, known as the medial patellofemoral ligament complex (MPFC), combining the MPFL and MQTFL into 1 construct (Figure 1).

**FIGURE 1:**
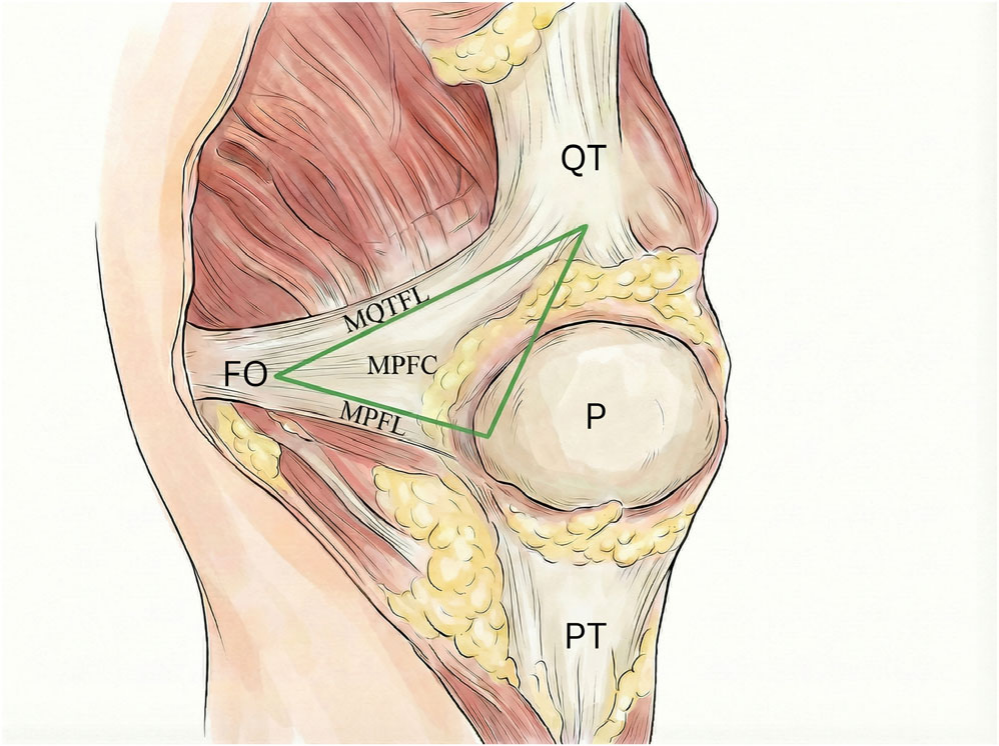
Image showing the medial patellofemoral complex anatomy, with fixed points being at the QT, PT, and the FO. This allows attachment of the respective arms of the complex, namely, the MPFL and the MQTFL. These arms form the MPFC. (FO, femoral origin; MPFC, medial patellofemoral ligament complex; MPFL, medial patellofemoral ligament; MQTFL, medial quadriceps tendon femoral ligament; P, proximal; PT, patellar tendon; QT, quadriceps tendon.)

There are multiple techniques in the literature focusing on either isolated MPFL or isolated MQTFL reconstruction, including bony fixation or all soft tissue.^4^, ^5^ The technique in this paper describes an anatomical reconstruction of the MPFC, combining 2 distinct MPFL and MQTFL limbs into 1 anatomical construct, using a gracilis allograft, with bony fixation at the MPFC midpoint on the patella, isometric bony fixation in the femur, and soft‐tissue stabilization on the quadriceps tendon. This technique is primarily designed for skeletally mature individuals and allows for dynamic tensioning of the graft.

## SURGICAL TECHNIQUE

The technical aspects of the surgical technique are shown in Video 1.

### Indications

This technique is indicated for skeletally mature patients with recurrent instability of the patella with demonstrable MPFC tear or laxity on history/examination and confirmed attenuation or tear on magnetic resonance imaging. Magnetic resonance imaging is also useful for elucidating any bony features of maltracking such as increased tibial tubercle‐trochlear groove distance, severe trochlear dysplasia, or malrotation as isolated soft tissue reconstruction without addressing underlying maltracking can lead to deleterious outcomes.^6^, ^7^ Additionally, a plain radiograph of the knee, particularly a lateral view at 30° of flexion, is most useful for delineating patella alta. The MPFC reconstruction technique may be indicated as an isolated procedure in certain patients, provided that a structured workup has shown no coronal malalignment (on long‐leg standing plain radiographs) or axial plane rotational deformity using either a 3‐dimensional computed tomography tracking study or a computed tomography rotational profile to ascertain for over femoral anteversion or external tibial torsion.

### Preoperative Preparation

The patient is placed in the supine position, with a side support and a foot support at 45° of knee flexion. A diagnostic arthroscopy can be performed to look for any intra‐articular pathology, such as a plica, or to perform an arthroscopic lateral release, if required. A list of the equipment needed is provided in Table 1.

**TABLE 1:** Equipment Setup

**Equipment**
Presutured gracilis allograft, 5 mm in diameter and 260 mm in length
PEEK knotless SwiveLock anchor loaded with a No. 2 FiberWire (SwiveLock, Arthrex, Naples FL)
No. 0 fibreloop suture (FibreLoop, Arthrex)
No. 2 fibrelink suture (FibreLink, Arthrex)
2.4‐mm guidewire for 6‐mm cannulated reamer
1.1‐mm nitinol wire
6 mm × 30 mm PEEK interference screw (FastThread PEEK Interference Screw, Arthrex)

PEEK, polyether ether ketone.

### Patellar Fixation

A 5‐ to 10‐cm midline incision is made centered on the junction of the quadriceps insertion onto the patella. Diathermy is used to expose the midpoint of the MPFC insertion which has been shown to be isometric and proximal to the junction of the proximal and middle thirds of the articular patella (33% of the patellar articular length) (Figure 2).^8^, ^9^

**FIGURE 2:**
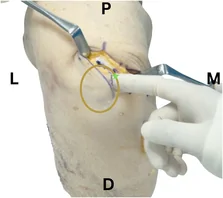
Cadaveric right knee A‐P view. Showing the insertion point (green star) of the MPFL onto the patella (outlined gold circle). This is shown to be isometric and P to the junction of the P and middle thirds of the articular patella (33% of the patellar articular length). (A‐P, antero‐posterior; D, distal; L, lateral; M, medial; MPFL, medial patellofemoral ligament; P, proximal.)

A 2.4‐mm guidewire is passed at this point, coming out on the lateral side of the patella. Care should be taken not to violate either the articular surface of the patella or the dorsal tension surface (Figure 3). A 5‐mm reamer is used to ream to a horizontal depth of 22 mm. This provides a short and small‐diameter patellar aperture. A sutured end of the gracilis allograft is threaded through the guidewire eyelet, and the wire is passed out of the lateral side of the patella completely to deliver the sutures with it. The sutures attached to the allograft, now on the lateral side of the patella, are used to pull the graft into the newly created medial patellar aperture (Figure 4). At this stage, an interference fixation is performed using the PEEK (polyether ether ketone) 4.75‐mm swivelock anchor (SwiveLock, Arthrex, Naples, FL), with care being taken to keep the graft taught at both ends to avoid twisting the graft into the aperture (Figure 5). At this stage, layer 2 is identified (where the MPFC runs anatomically), and an opening is made with a hemostat to allow passing of the allograft through this space to the eventual femur insertion point.

**FIGURE 3:**
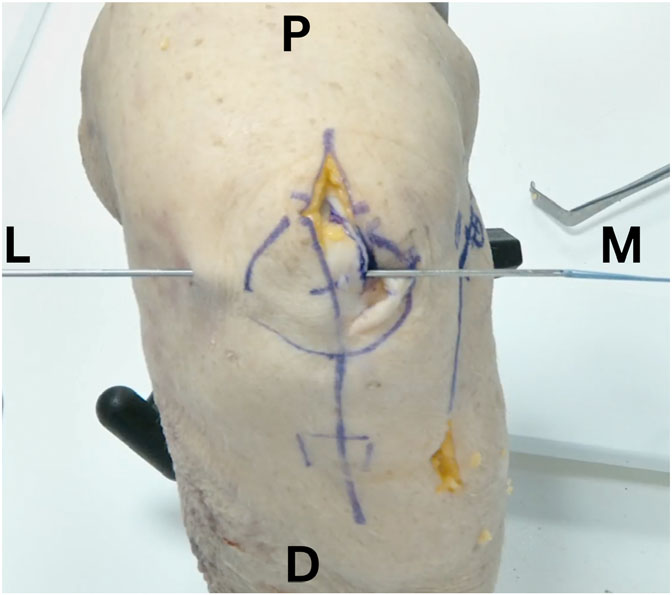
Cadaveric right knee A‐P view. A 2.4‐mm guidewire is passed through the insertion point at the patella, coming out on the lateral side of the patella. Care should be taken not to violate either the articular surface of the patella or the dorsal tension surface. (A‐P, antero‐posterior; D, distal; L, lateral; M, medial; P, proximal.)

**FIGURE 4:**
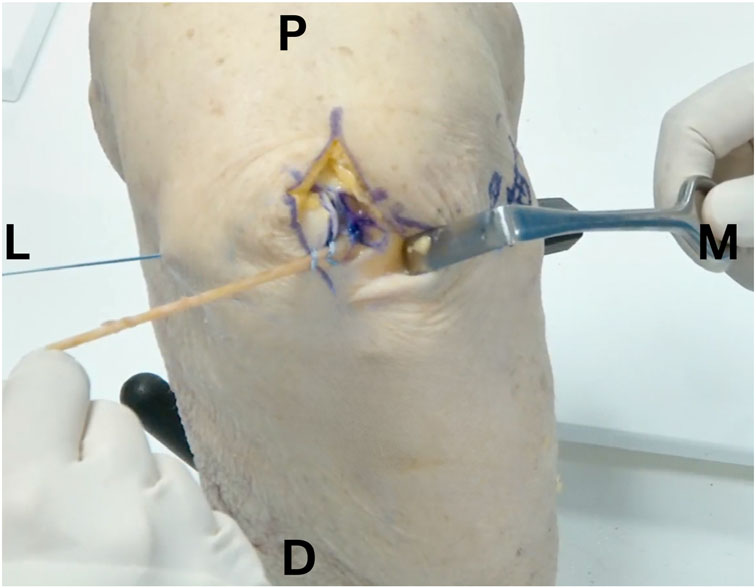
Cadaveric right knee A‐P view. The sutures attached to the allograft, now on the lateral side of the patella, are used to pull the graft into the newly created medial patellar aperture. (A‐P, antero‐posterior; D, distal; L, lateral; M, medial; P, proximal.)

**FIGURE 5:**
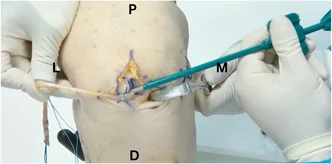
Cadaveric right knee A‐P view. Interference fixation is performed using the PEEK swivelock anchor, with care being taken to keep the graft taught at both ends to avoid twisting the graft into the aperture. (A‐P, antero‐posterior; D, distal; L, lateral; M, medial; P, proximal; PEEK, polyether ether ketone.)

### Femoral Fixation

After identifying Schottle's point on the femur under lateral fluoroscopy, a separate medial skin incision is made directly over this and down onto the bone.^10^ The MPFC footprint on the femur is posterior to the medial epicondyle and distal to the adductor tubercle. A bony depression can often be palpated at this point (Figure 6). After the identification of this point once on the bone using a true lateral view under fluoroscopy, a 2.4‐mm guideline is driven in a proximal and anterior position, exiting anterolaterally in the femur and the skin (Figure 7).

**FIGURE 6:**
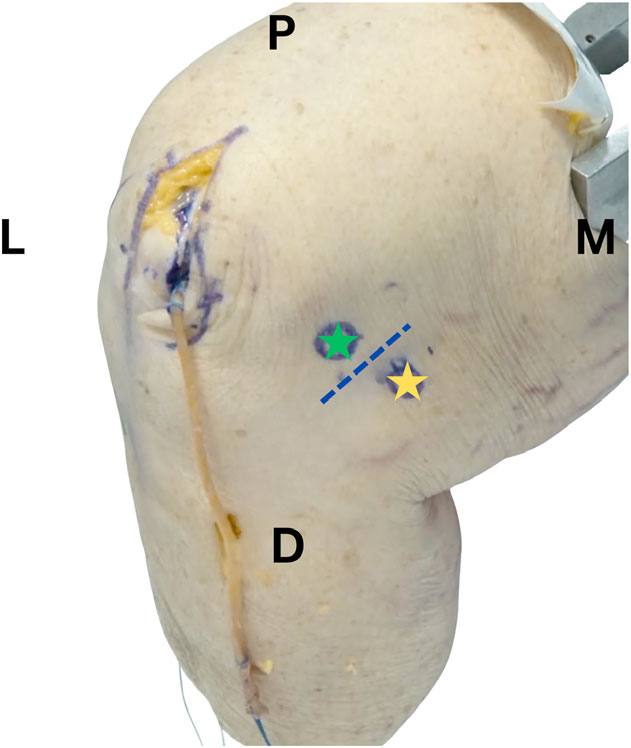
Cadaveric right knee medial view. Anatomic landmarks for medial incision to identify Schottle's point. The MPFC footprint on the femur is posterior to the medial epicondyle (green star) and distal to the adductor tubercle (yellow star). A bony depression can often be palpated at this point. (D, distal; L, lateral; M, medial; MPFC, medial patellofemoral ligament complex; P, proximal.)

**FIGURE 7:**
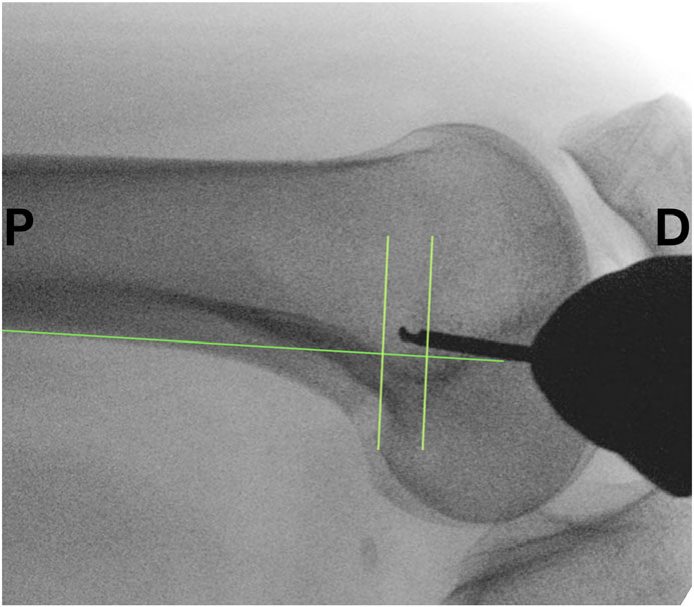
Fluoroscopic true lateral view of the distal femur. A 2.4‐mm guideline is driven in a proximal and anterior position exiting anterolaterally in the femur and through the skin. (D, distal; P, proximal.)

Over‐reaming to a depth of 45 mm is then undertaken with a 7‐mm reamer. A No. 2 fibrelink (FibreLink, Arthrex) passing suture is pulled through laterally, leaving the loop at the medial aperture created in the femur. At this stage, the graft is passed through layer 2 and the loop, with the loop centered on the midpoint of the graft (Figure 8).

**FIGURE 8:**
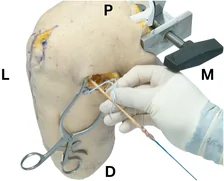
Cadaveric right knee medial view. A No. 2 fibrelink passing suture is pulled through laterally, leaving the loop on the medial aperture created on the femur. At this stage, the graft is passed through layer 2 and the loop, with the loop centered on the midpoint of the graft. (D, distal; L, lateral; M, medial; P, proximal.)

### Quadriceps Insertion

Attention is turned back to the midline incision. A partial thickness incision is made over the junction of the medial and central thirds of the quadriceps insertion (Figure 9). A right‐angled clamp is used to create a passage for the remaining free end of the graft. At this point, with the graft having been fed through the fibrelink (FibreLink, Arthrex), the remaining free end of the allograft is retrieved again at the level of the patella through layer 2. The graft is passed through the midline partial thickness incision that has been newly created using the right‐angled clamp. The free end of the allograft can now be sutured with interrupted No. 1 Vicryl sutures onto the quadriceps and patellar periosteum and/or into the interval of the medial patellar incision (Figure 10). Care must be taken to leave enough slack in the graft at the level of the femur for passage into the femoral footprint and dynamic tensioning.

**FIGURE 9:**
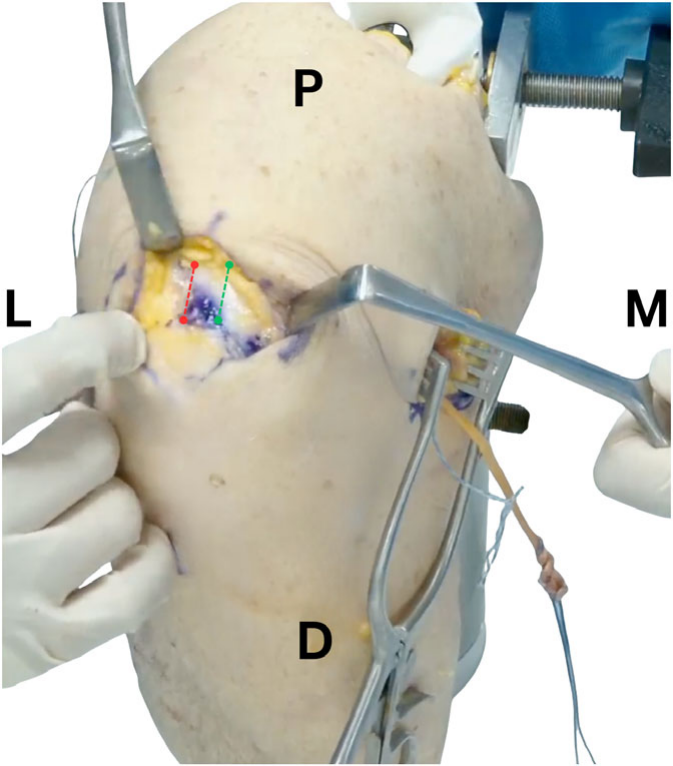
Cadaveric right knee A‐P view. A partial thickness incision is made over the junction of the medial and central thirds of the quadriceps insertion (red dashed line). The medial border of the quadriceps insertion is denoted by the green dashed line. (A‐P, antero‐posterior; D, distal; L, lateral; M, medial; P, proximal.)

**FIGURE 10:**
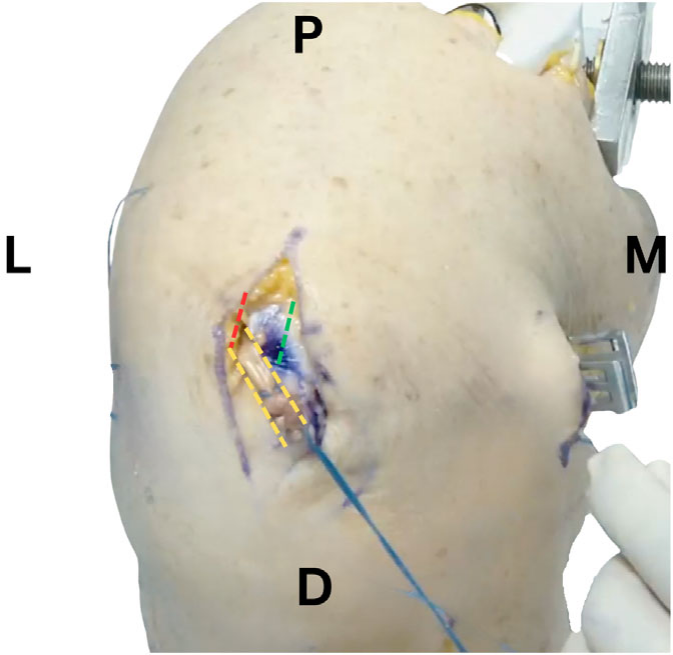
Cadaveric right knee A‐P view. The graft is passed through the midline partial thickness (yellow dashed line) incision that has been newly created using the right‐angled clamp. The free end of the allograft can now be sutured with interrupted No. 1 Vicryl sutures onto the quadriceps and patellar periosteum and/or into the interval of the medial patellar incision. (A‐P, antero‐posterior; D, distal; L, lateral; M, medial; P, proximal.)

### Tensioning and Femoral Fixation

Now that both ends of the graft have been secured (patellar MPFC midpoint and the MQTFL on the quadriceps), a loop of graft should remain within the No. 0 fibreloop passing suture (FibreLoop, Arthrex). Using the free end of the fibrelink (FibreLink, Arthrex) laterally, pulling on this draws the central aspect of the allograft which has been fed into the loop into the femoral aperture (Figure 11). Dynamic tensioning can occur on the patella by adjusting the pulling force on the fibrelink (FibreLink, Arthrex) suture. The authors prefer half a quadrant of lateral deviation at 45° of knee flexion as described above, and the graft is tensioned and secured at this angle because it has previously been shown that this angle correlates with the patella being constrained within the trochlea and prevent overtensioning of the graft.^11^ The graft is secured by passing a 1.1‐mm nitinol guidewire into the femoral aperture under the looped graft, followed by a 6‐mm‐diameter × 30‐mm‐length PEEK interference screw (FastThread PEEK Interference Screw, Arthrex) to fix the graft in situ (Figure 12).

**FIGURE 11:**
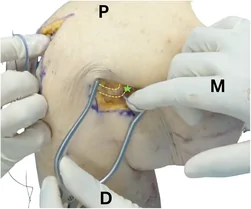
Cadaveric right knee medial view. Using the free end of the fibrelink laterally, pulling on this draws the central aspect of the allograft (yellow dashed line) which has been fed into the loop into the femoral aperture (green star). (D, distal; M, medial; P, proximal.)

**FIGURE 12:**
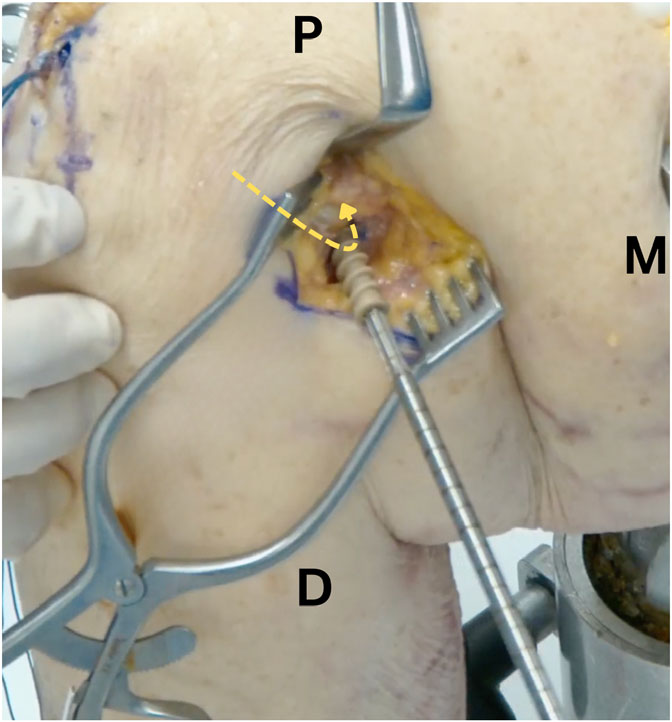
Cadaveric right knee medial view. A 1.1‐mm nitinol wire is passed into the femoral aperture under the looped graft, followed by a 7‐mm‐diameter × 30‐mm‐length PEEK interference screw to fix the graft in situ. Yellow dashed arrow denotes trajectory of the graft into the femoral aperture. (D, distal; M, medial; P, proximal; PEEK, polyether ether ketone.)

## DISCUSSION

Our understanding of the medial patellofemoral complex has evolved over the last decade, with an increasing focus on the MQTFL and its role in patellar stability. Evolving from the copious data and techniques with regard to MPFL reconstruction, it is important to build on emerging anatomical knowledge by following on with anatomical reconstructions. Additionally, it is important that a dynamic graft tensioning technique is used given the varying literature surrounding the optimal tensioning angle,^12^ and therefore, this provides the operating surgeon with the flexibility to tension according to their evidence‐based principles.

This technique accurately and anatomically replicates our current understanding of the soft‐tissue medial patellofemoral restraint to lateral patellar instability by reconstituting the individual arms of the medial patellofemoral complex. This is a single‐loop technique that combines both the bony and soft‐tissue elements of the MPFC. Several advantages can be seen with this technique (Table 2): (1) the anatomical nature of the reconstruction; (2) single small‐diameter and short patellar aperture, without broaching either the lateral cortex or the anterior tension‐sided cortex, negating the need for bicortical large apertures and reducing fracture risk; (3) dynamic tensioning allowing for intraoperative balancing of tension and reducing the risk of overtensioning in flexion or undertensioning causing laxity; (4) the security of having an additional “backup” limb of the MPFC should one of the limbs fail/stretch; (5) the minimally invasive nature in terms of skin incisions (2 incision techniques); and (6) the minimal equipment needed for this technique. Potential pitfalls of this technique lie in Table 3: (1) not drilling sufficient femoral aperture depth, thereby leading to early bottoming out of the graft and undertensioning; (2) any inaccuracy of the femoral footprint will lead to anisometry of both the MPFL and MPFC limbs; and (3) the reliance on a patellar aperture with patellar drilling being associated with fracture risk.^13^

**TABLE 2:** Advantages and Disadvantages

**Advantages**	**Disadvantages**
Anatomically reconstitutes both limbs of the medial patellofemoral complex (MPFL and MQTFL) in a single continuous construct	Relies on a patellar tunnel; any patellar drilling carries an inherent risk of patellar fracture
Single small‐diameter (5 mm), short (22 mm) patellar aperture that avoids breaching the lateral or anterior (dorsal tension‐sided) cortex, obviating large bicortical tunnels, and reducing fracture risk	Use of allograft adds material cost
Dynamic, adjustable graft tensioning permits intraoperative balancing and reduces the risk of overtensioning in flexion or residual laxity from undertensioning	Femoral fixation requires intraoperative fluoroscopy to reliably identify Schottle's point
A redundant backup limb provides ongoing restraint should either limb of the complex stretch or fail	New technique comes with a learning curve
Minimally invasive, 2‐incision approach	Primarily indicated for skeletally mature patients; drilling risks the physis in immature knees
Requires minimal specialized equipment	Does not address bony or rotational malalignment; adjunctive procedures are needed when these are present

MPFL, medial patellofemoral ligament; MQTFL, medial quadriceps tendon femoral ligament.

**TABLE 3:** Pearls and Pitfalls

**Pearls**	**Pitfalls**
Place the patellar tunnel at the isometric MPFC midpoint—proximal to the junction of the proximal and middle thirds of the articular patella (approximately 33% of articular length)	Violating the patellar articular surface or the dorsal tension‐sided cortex during drilling increases fracture risk
Keep the graft taut at both ends during swivelock interference fixation to prevent it twisting within the patellar aperture	Inaccurate femoral footprint placement produces anisometry of both the MPFL and MQTFL limbs
Confirm Schottle's point on a true lateral fluoroscopic view before drilling the femur	Insufficient femoral aperture depth causes early graft bottoming out and undertensioning
Over‐ream the femoral tunnel to 45 mm depth to accommodate the looped graft and avoid bottoming out	Overtensioning in flexion can overload or capture the medial patellar facet
Leave adequate graft slack at the femur for passage into the footprint and for dynamic tensioning	Failure to pass the graft within layer 2 disrupts the anatomic course of the complex
Tension with the graft secured at 45° of flexion to a half‐quadrant of lateral patellar glide, keeping the patella constrained within the trochlea	Skipping the preoperative malalignment and rotational workup risks isolated soft‐tissue reconstruction failure

MPFC, medial patellofemoral ligament complex; MPFL, medial patellofemoral ligament; MQTFL, medial quadriceps tendon femoral ligament.

## DECLARATION OF GENERATIVE AI AND AI‐ASSISTED TECHNOLOGIES IN THE WRITING PROCESS

During the preparation of this work, the authors used OperativeInk.com to animate Figure 1 for the clarity of the diagram from a cadaveric image.

## DISCLOSURES

The authors (K.A‐H., X.L.) declare the following financial interests/personal relationships which may be considered potential competing interests: K.A‐H. reports payment or honoraria for lectures, presentations, speakers bureaus, manuscript writing, or educational events from Joint Operations consulting fees (not relevant to this paper) and support for attending meetings and/or travel from Joint Operations (support for attending AAOS 2026). X.L. reports consulting fees from FH Orthopedics.
